# How Do Health Professionals Maintain Compassion Over Time? Insights From a Study of Compassion in Health

**DOI:** 10.3389/fpsyg.2020.564554

**Published:** 2020-12-29

**Authors:** Sofie I. Baguley, Vinayak Dev, Antonio T. Fernando, Nathan S. Consedine

**Affiliations:** ^1^Department of Psychological Medicine, University of Auckland, Auckland, New Zealand; ^2^Taylor Centre, Auckland District Health Board, Auckland, New Zealand

**Keywords:** compassion, physician—patient communication, emotion regulation, healthcare [MeSH], emotion, health

## Abstract

Although compassion in healthcare differs in important ways from compassion in everyday life, it provides a key, applied microcosm in which the science of compassion can be applied. Compassion is among the most important virtues in medicine, expected from medical professionals and anticipated by patients. Yet, despite evidence of its centrality to effective clinical care, research has focused on compassion fatigue or barriers to compassion and neglected to study the fact that most healthcare professionals maintain compassion for their patients. In contributing to this understudied area, the present report provides an exploratory investigation into how healthcare professionals report trying to maintain compassion. In the study, 151 professionals were asked questions about how they maintained compassion for their patients. Text responses were coded, with a complex mixture of internal vs. external, self vs. patient, and immediate vs. general strategies being reported. Exploratory analyses revealed reliable individual differences in the tendency to report strategies of particular types but no consistent age-related differences between older and younger practitioners emerged. Overall, these data suggest that while a range of compassion-maintaining strategies were reported, strategies were typically concentrated in particular areas and most professionals seek to maintain care using internal strategies. A preliminary typology of compassion maintaining strategies is proposed, study limitations and future directions are discussed, and implications for the study of how compassion is maintained are considered.

## Introduction

Compassion is one of the most important virtues in medicine, expected from medical professionals and anticipated by patients (Frampton et al., 2013; Fernando and Consedine, 2014a). Despite evidence of its centrality to effective clinical care, most research has focused on compassion fatigue and burnout (Fernando and Consedine, 2014a, b), neglecting the study of compassion itself (Fernando and Consedine, 2014a; Strauss et al., 2016). More recently, researchers have identified several factors or barriers that may interfere with compassion in healthcare settings (Fernando and Consedine, 2014b). Although this is a promising digression from the compassion fatigue framework, studies have not yet investigated how medical professionals *maintain* compassion for their patients. To bridge this gap in an area in which compassion research has serious implications, this study was designed to preliminarily characterize and categorize the types of strategies medical professionals use to maintain compassion for their patients.

In beginning to consider how medical professionals may maintain compassion, it is useful to briefly clarify the construct. As in other areas of study, there is ongoing debate about the nature of compassion, both in medicine and in other areas of study. In medicine, compassion is broadly thought to consist of two core elements—a deep awareness and willingness to gain knowledge of an individual’s suffering *and* a desire to relieve the suffering (Chochinov, 2007). Although more differentiated views (e.g., Strauss et al., 2016) suggest that compassion may incorporate up to five elements—recognizing suffering, understanding the universality of human suffering, feeling for the person suffering, tolerating uncomfortable feelings, and motivation to act/acting to alleviate suffering—the strong desire to alleviate suffering appears to be the key characteristic that differentiates compassion from related prosocial responses like empathy, concern, and sympathy (Goetz et al., 2010; Sinclair et al., 2016). In relation to the present study, recognizing that compassion is a complex and differentiated construct means we must think broadly when seeking to characterize the strategies medical professionals report using as a means to maintain it.

In considering the need for such research more fully, it is worth recalling how compassion is important to health systems. First, compassion and related states are important through their associated positive patient outcomes such as reductions in anxiety (Fogarty et al., 1999), improved patient satisfaction (Lown et al., 2011), strengthened physician-patient relationships (Fogarty et al., 1999), lower PTSD following health emergencies (Moss et al., 2019), and better health outcomes (Post, 2011; Del Canale et al., 2012); research suggests that doctors who are more compassionate provide more meticulous care (Trzeciak et al., 2017). Second, compassion is associated with positive outcomes for physicians, including higher job satisfaction (Gleichgerrcht and Decety, 2013, 2014) and job retention (Sinclair et al., 2016). Neurological studies suggest a clear overlap between pleasure and compassion activation in physicians (Klimecki et al., 2014), which may buffer or counteract stress pathways (Abdulghani et al., 2011; Trzeciak et al., 2017). Three, compassion is a professional obligation and a patient right (Bramley and Matiti, 2014), legislatively mandated in most countries (Paterson, 2011). Finally, although data are scanty, compassion may be associated with financial benefits for health care systems, via lower absenteeism, fewer malpractice lawsuits, and fewer medical errors (Trzeciak et al., 2017). In sum, compassion is critical to effective and sustainable healthcare environments implying that identifying the strategies medical professionals report using to maintain compassion may be of benefit.

A small prior literature has examined the ways by which healthcare professionals attempt to connect with their patients, although studies are a few and remain restricted to nursing and psychology samples. A recent qualitative study of mental health nurses (*n* = 13) and patients receiving care (*n* = 7) suggested that asking patients questions and reflecting on their own difficulties were the most frequently reported strategies when attempting to cultivate empathy (Gerace et al., 2018). More broadly, psychological writings suggest that mindfulness meditation (Block-Lerner et al., 2007; Shapiro and Izett, 2008), self-compassion (Morgan and Morgan, 2005; Gilbert, 2009; Neff, 2012; Fulton, 2018), and connecting with patients are seen as facilitating empathy and rapport (Bibeau et al., 2016). As noted, however, while empathy may provide a path to compassion, it is distinct. Compassion in healthcare is distinct insofar as it is professionally mandated and repeated; although strategies may be shared, healthcare contexts represent a particular challenge to care.

To this point, however, how healthcare professionals maintain compassion and the strategies they use remains unknown—studies have focused on compassion fatigue (Van Mol et al., 2015; Nolte et al., 2017; Sinclair et al., 2017) and burnout (Potter et al., 2010). However, compassion fatigue is a problematic construct (Fernando and Consedine, 2014a; Ledoux, 2015), in part because it does not capacitate interventions to improve or maintain compassion. The notion of fatigue tends to imply that healthcare workers have a reservoir of compassion which, when depleted, leads to services being provided without care (Fernando and Consedine, 2014a). Empirically, however, while this view implies that compassion should be reduced over time (Fernando and Consedine, 2014a), empirical work suggests the opposite; compassion fatigue is reliably *lower* in more experienced workers (Shanafelt et al., 2009; Potter et al., 2010; Berger et al., 2015; Dasan et al., 2015). Although age is conflated with differences in seniority, autonomy, and the like, such findings imply that older, more experienced physicians may have developed strategies to manage their ability to care. Therefore, a second aim of this study was to test for age-related differences in the normative strategies used to maintain compassion. Possible differences in the strategies used by more experienced professionals may inform how medical trainees might be instructed in ways that facilitate the maintenance of compassion.

Other recent studies seeking areas in which interventions might be developed have examined the *barriers* to compassion in healthcare. Researchers in this area have developed the Transactional Model of Compassion (Fernando and Consedine, 2014a), which views compassion as a dynamic process influenced by factors from four key areas: the physician, patient and family, clinical context, and environmental and institutional factors. Evidence gathered within this framework suggests that barriers vary in predictable ways across healthcare disciplines (Fernando and Consedine, 2017) and lower with more experience (Dev et al., 2019). Again, however, while this work has deepened our understanding of what *interferes with* compassion in health contexts, it focused on barriers and is readily supplemented by studies of the processes used to *enhance* compassion.

## Study Objectives

Given evidence for compassion’s centrality to effective clinical care and the benefits it holds for patients, practitioners, and the healthcare system, the objectives of the current study were to (a) preliminarily identify and characterize the strategies medical professionals use to maintain compassion for their patients, and (b) explore potential developmental variation in such strategies.

In the absence of prior work circumscribing the processes that health professionals use to maintain compassion in clinical practice, making specific predictions regarding possible strategies was not possible. However, it seemed reasonable to expect that professionals would report a range of strategies encompassing general self-care and self-management, as well as strategies that specifically involve changing the way they think about themselves, their roles, and their patients. Strategies seemed likely to encompass a mixture of approaches that were oriented to the self vs. concerned with the patient as well as those that were more internally vs. externally focused. Some strategies might be very “immediate” and thus suited to deployment during clinical encounters while others may well be more “distal” and reflect more general self-maintenance strategies that (subjectively) allow for compassion to be sustained over time. As noted, as a secondary interest, we also investigate the possibility that the tendency to report different types of strategies are used will vary developmentally, with younger vs. older practitioners potentially being more and less likely to employ different types of strategies.

## Materials and Methods

### Study Design and Setting

Permission to conduct the present study was obtained from The University of Auckland Human Participants Ethics Committee (Approval Number: 022709). Health professionals (*N* = 300), registered to attend the Compassion in Healthcare Conference in New Zealand in March 2019, were recruited to the study via email 2 weeks prior to the event. Participants were sent an email invitation containing a link to a description of the study and a 5-min questionnaire. The questionnaire included five demographic questions and three open-ended questions related to compassionate processes and behaviors (see Supplementary Appendix 1). Participants who completed the online questionnaires were invited to enter a draw to win one of two $100 gift vouchers. To preserve anonymity in the sample, minimal identifying data were acquired and contact information needed for the prize draw was not linked to primary responses.

### Participants

Participants (*N* = 151, 50.33% of invitees) responded to the study invitation. Of 151, 57.62% were physicians, 19.87% were nurses, 5.96% were psychologists, and 16.55% other health professionals. Across the sample, 84.11% were female. The mean age of physicians was 46.32 years, of nurses was 52.77 years, of psychologists was 48.47 years, and of other professionals 48.64 years. Participants reported being born in New Zealand (51.66%), the United Kingdom (20.53%), Europe (10.60%), Asia (3.98%), and elsewhere (13.23%; see Table 1).

**TABLE 1:** Demographic characteristics of the health professional sample.

Variable	*n* (% or SD)
Ethnicity	
New Zealander	78 (51.66%)
British	31 (20.53%)
European	16 (10.60%)
Asian	6 (3.98%)
Other	19 (13.23%)
Age (in years)	
Physicians	46.32 (± 11.58)
Nurses	52.77 (± 11.79)
Psychologists	48.47 (± 11.79)
Other	48.64 (± 10.86)
Gender	
Females	127 (84.10%)
Males	24 (15.89%)
Profession	
Physicians	87 (57.62%)
Nurses	30 (19.87%)
Psychologists	9 (5.96%)
Other	25 (16.55%)

### Variables and Data Measurement

#### Background Characteristics

Participants were asked to report gender (male/female), age, and country of birth. Regarding professional practice, participants were asked to describe their main type of clinical practice; responses were coded as: “medicine/surgery,” “nurse,” “psychologist,” and “other.” Descriptive characteristics are summarized in Table 1 below.

#### Coding Self-Reported Strategies to Maintain Compassion

In this preliminary investigation of the processes by which medical professionals maintain compassion for their patients, respondents were asked two, open-ended questions. The target question stated that: “Sustaining compassion over time can be difficult. How do you maintain compassion for your patients inside yourself?” A second question asked whether “There are (*sic*) particular things you do or think about when seeking to maintain compassion?” Responses to the first question were coded using a system developed for the study and is described below.

The development of the coding system was an iterative process that combined top-down theoretical considerations with bottom-up categories based in the data themselves. Ultimately, coding categories were based on three considerations: (a) the five elements of compassion proposed by Strauss et al. (2016) and the influences on care posited in the Transactional Model of Physician Compassion (Fernando and Consedine, 2014a), (b) the content of the responses from participants, and (c) guidance from academics and clinicians with expertise in communication and compassion, including the authors (NC, AF). Ultimately, six content global categories were coded: connecting with the patient, common humanity, empathy, tolerance of psychological discomfort, mindfulness, and general self-care/maintenance, together with an “uncodable” category. As is typical in research of this kind, the six content categories acted as supraordinate classes of compassion-maintaining strategy and were further sub-coded to create greater differentiation. After several iterative phases of development, the resultant system appeared as summarized in Table 2.

**TABLE 2:** Final coding system used to score self-reported compassion maintaining strategies in healthcare professionals.

Supraordinate code	Sub-codes	Description	Example
1. Connection with patient	1a. Communication (CP1)	Reference made to the use of communication skills as a means to maintain compassion for patients.	“I utilize open-ended questions.”
	1b. Active listening (CP2)	Reference made to using active listening to maintain compassion.	“I put down my pen and listen.”
2. Common humanity	2a. General common humanity (CH1)	Reference made to the shared or common nature of being human.	“I acknowledge that we are more similar than different.”
	2b. Common humanity of suffering (CH2)	Response specifically includes reference to the commonality of *suffering*.	“I remind myself that all people suffer.”
3. Empathy	3a. Openness to experience of patients (EP1)	*Explicit* reference made to remaining “open” to patients or text indicated a more general openness to the experience of others.	“deliberately or consciously open,” “open to them.”
	3b. Cognitive empathy (EP2)	Reference made to taking the patient’s perspective.	“I try to imagine their situation from their perspective and put myself in their shoes.”
	3c. Affective empathy (EP3)	Reference made to *emotionally* connecting with patient distress.	“I feel moved by the person suffering.”
4. Tolerance of psychological discomfort	4a. Tolerance of psychological discomfort (TD)	References *internal or external* psychological processes aimed at increasing the ability to tolerate or manage distress, difficult feelings, or discomfort.	“I remind myself it is not their fault they are being difficult and take a breather from the situation.”
5. Mindfulness	5. Mindfulness	Reference made to mindfulness as a tool/strategy and/or a way of being/state of mind to maintain compassion for patients.	“I practice mindfulness.”
6. Self-care/maintenance	6a. Recognizing limits (SC1)	Reference made to recognition of personal or professional limits.	“I acknowledge that I am human and cannot fix everything.”
	6b. Supervision (SC2)	Reference made to using supervision to maintain compassion.	“I get regular supervision,” or “see a clinical psychologist.”
	6c. Exercise (SC3)	Reference made to using exercise or physical activity as a means of self-care.	“I go to the gym or for a run.”
	6d. Refresh (SC4)	Reference made to taking time out to refresh and look after wellbeing.	“I take a holiday when I need.”
	6e. Socialization (SC5)	Reference made to engaging in socialization for self-care/maintenance.	“Catching up with people close to me.”
	6f. Prevent burnout (SC6)	Reference made to limiting work schedules to prevent burnout or exhaustion.	“I ensure I take 2 days off each week.”
	6g. Praying (SC7)	Reference made to use of prayer as a means to maintain compassion for patients.	“I pray.”
	6h. Going to church (SC8)	Reference made to the act of going to church or a religious establishment.	“I visit my place of worship.”
	6i. Meditation (SC9)	Reference made to using meditative practices.	“I practice meditation.”
	6j. Other physical and mental self-care (SC10)	Reference to other self-care processes not captured by the categories above.	

#### Coding Procedure

All 151 responses were coded independently by two coders (SB and VD) using the coding scheme developed for the study. Given the open-ended nature of the prompting question, codes were not mutually exclusive; responses could score concurrently in multiple categories and/or subcategories. For each category or subcategory, responses were coded on a present/absent basis. When responses were not scored for any of the six primary content codes they were coded in category (7), as “uncodable.”

Following independent coding, inter-rater reliability was assessed. Overall agreement between raters was κ (Cohen’s Kappa) = 0.70 with categories of strategies ranging from κ = 0.61 to κ = 0.93. The independent coding resulted in disagreement on 4.39% of 3,222 codes. Discrepencies were resolved between the two coders by discussing each individual code and agreeing on the category of best fit. Discrepancies were coded in favor of the first coder 39% (*n* = 57) and the second coder 61% (*n* = 89) of the time.

## Results

In line with the aims of this report, responses were analyzed in three steps—descriptive characterization of individual strategies and strategy types, examination of the extent to which individuals systematically preferred particular types of strategy (an examination of proportions), and developmental analyses.

### Descriptive Characterization of Strategies

Of the 151 healthcare professionals in the sample, 52.79% reported utilizing self-care behaviors, 18.44% reported using empathy, 13.41% common humanity, 5.87% connection with patient, 4.19% tolerance of discomfort, and 3.07% reported using mindfulness as a means to maintain compassion. Fewer than 3% of responses were uncodable (see Table 3). Descriptively, 60.06% of the responses indexed reports of using self-focused strategies as a means to maintain compassion while 37.71% reported on patient-focused strategies. As previously, the remaining 2.23% of responses could not be classified. Other examinations showed that 55.87% of the sample reported internally focused strategies while 22.35% of the responses reported use of external strategies; the remaining 21.78% of responses could not be classified as either internal or external focused. Finally, 55.86% of strategies were classified as general and 41.91% as “immediate” strategies, more likely used in the present moment during patient consultation.

**TABLE 3:** Descriptive breakdown of self-reported strategies to maintain compassion.

Strategy	Number of participants reporting use of strategy (%)
Connection with patient	21 (5.87%)
Common humanity	48 (13.41%)
Empathy	66 (18.44%)
Tolerance of discomfort	15 (4.19%)
Mindfulness	11 (3.07%)
Self-care behaviors	189 (52.79%)
Uncodable	8 (2.23%)

*Because responses could be scored in multiple categories, percentages may not total 100.*

### Frequency of Different Strategy Types

Second, we considered how the frequency of strategy use was distributed across different *types* of strategy and whether individuals manifest any preference for particular classes of strategy. To minimize Type 1 error inflation, individual codes were recoded into conceptually meaningful categories. So, for the self-other strategy contrast, strategies were regrouped into self-focused (distress tolerance, mindfulness, and self-care codes) vs. strategies which focused attention toward the patient (connection with patient, common humanity, empathy). Second, to examine differences in the use of internal vs. external strategies, strategies were regrouped into those involving internal mental processes (connection with patient, common humanity, empathy, distress tolerance processes, mindfulness, and self-care codes SC1, SC7, and SC9) and strategies involving use of external or behavioral processes (self-care codes SC2, SC3, SC4, SC5, SC6, and SC8). Finally, as analyses progressed, it became clear that some strategies might also be usefully classified as immediate while some were more general. To capture this possible distinction, strategies were regrouped into those occurring within the clinical context (connection with patient, common humanity, empathy and distress tolerance) vs. strategies that were not of exclusive/primary relevance to the immediate clinical context and/or were not limited by time (mindfulness, and self-care).

### Questions Regarding Individual Differences in Strategy Use

To facilitate interpretation and enable an examination of whether individuals typically reported strategies of particular kinds, these scores were then proportionalized. The raw proportions of self vs. other, and immediate vs. general, and internal vs. external were recoded into 0 (strategy type not reported), 1 (a proportion between 0 and 1) and 2 (100% of reported strategies in that class). Chi-square analyses were then used to examine the proportions of self vs. other-focused strategies to maintain compassion.

**FIGURE 1:**
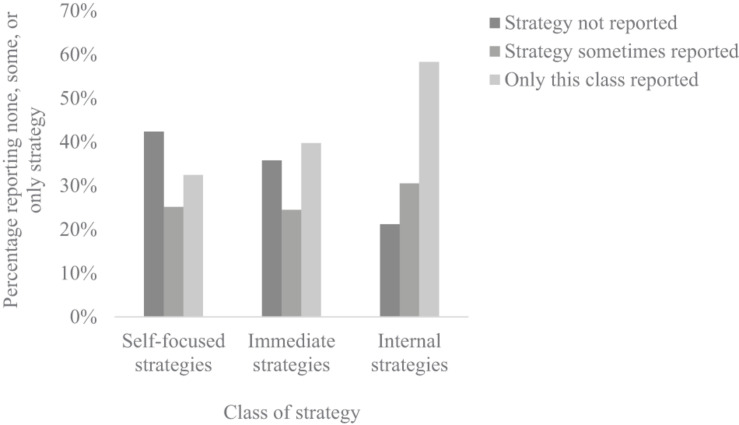
Bar graph demonstrating proportions of the sample reporting none, some, or only self-focused, immediate, and internal compassion maintaining strategies.

Descriptively, 42.38% reported no self-focused strategies, 25.17% reported some, and for 32.45% of the sample, all strategies were self-focused. Comparably, for the proportions of immediate vs. general strategies, 35.76% reported no immediate strategies, 24.50% reported a mixture of immediate and general strategies, and for 39.74% all reported strategies were immediate in nature. Finally, analyses of the proportion of internal strategies suggested that 21.19% reported no use of internal strategies, 20.53% reported a mixture of internal and external, and for 58.28% of the sample, all strategies were internal strategies to maintain compassion (see Figure 1 below).

### Possible Developmental Variation in Strategy Use

Given the trend toward lower compassion fatigue and lower barriers to compassion with age and the substantial covariation between age and clinical experience (Dev et al., 2019), the possibility that healthcare professionals of different ages might differentially report different strategies was examined. For these analyses, respondents were split into three groups based on the sample characteristics (i.e., 20–43 years, 44–54 years, and 55–77 years). In the interests of parsimony, we restricted our tests to the proportionalized aggregate scores (internal v. external, self v. other, and immediate v. general).

Somewhat in contrast to expectation, analyses revealed no age-related differences in the proportion of self vs. other-focused strategy use X^2^(2, *N* = 151) = 2.68, *p* = 0.613, nor were there age-related differences in the proportion of internal vs. external, X^2^(2, *N* = 151) = 1.93, *p* = 0.749, or immediate vs. general strategy use, X^2^(2, *N* = 151) = 0.945, *p* = 0.918. Because of some concern regarding cell sizes in the analyses of the portion of internal strategies, these proportions were further considered in a 3 (age group) by 2 (none vs. some or all) chi-square. Again, no difference was evident X^2^(2, *N* = 151) = 0.172, *p* = 0.918. Put simply, although individuals systematically varied in the extent to which they concentrated their strategies within particular classes (see Figure 1), there was no systematic developmental variation evident in the types of strategies reported in this sample.

## Discussion

In extending prior research, this report offers a first attempt at identifying and characterizing the strategies healthcare professionals reporting using to maintain compassion. While a few studies have examined how empathy might be facilitated in nursing (Gerace et al., 2018) and psychology samples (Bibeau et al., 2016), prior to this report no studies have specifically focused *on the maintenance of compassion in healthcare*. While this sample of healthcare professionals predominately reported employing self- over patient-focused strategies to maintain compassion, a range of strategies involving self-care, patient empathy, remembering patient humanity, and others were reported. In line with general expectations, participants varied in the extent to which strategies were concentrated in particular classes—people seem to have a “style” at least as measured in this way. However, developmental variation in strategy use was not evidenced. Below, these findings are discussed in further detail, positioned relative to existing work on compassion in health, some preliminary interpretations are given, and a provisional typology of strategies is offered.

### Characterizing *Types* of Compassion-Maintaining Strategies

As noted, prior studies of compassion in health have historically focused on the study of compassion fatigue (Van Mol et al., 2015; Nolte et al., 2017; Sinclair et al., 2017), a problematic construct that does little to illuminate potential interventions (Fernando and Consedine, 2014a). In contributing to work beyond the compassion fatigue and barriers frameworks, the current study provides a beginning to the process of examining the strategies by which the healthcare workers maintain compassion despite the serious challenges inherent to this environment.

Descriptively, the range of strategies subjectively experienced as enabling the maintenance of compassion was extensive. Participants reported the expected clustering of strategies involving the use of empathy and connecting to patients as well as tolerating their own discomfort, mindfulness, and reporting deliberately recalling the patients’ humanity. While empathy and connection are relatively widely studied (e.g., Amutio-Kareaga et al., 2017) and emotional skills (Zeidner et al., 2013) and mindfulness (e.g., Lim and DeSteno, 2016; Fernando et al., 2017) have also been studied in the context of compassion, the frequency with which common humanity was referenced is interesting.

Indeed, while they may reflect something of the particular sample employed in this study (see below), references to common humanity may suggest a relatively widespread understanding among healthcare professionals that recalling the commonality of suffering and/or consciously recalling that it is a *person* that is suffering helps maintain care. Exactly why perceptions of common or shared humanity are so central to compassionate responding is unclear, although although writings in the medical tradition suggest that stress and emotional upset in response to suffering may “trigger a safety ethic” in which compassion in replaced by a focus on patient *management* (Davin et al., 2018). Put simply, when the management of the patient’s condition (rather than the patient themselves) predominates, the patient’s humanity is lost and thus too a major component of compassion is absent. More broadly, it is notable that strategies involving mental changes of some kind accounted for approximately 45% of the reported strategies. Their commonality, coupled with the fact that such strategies are (a) potentially trainable and (b) do not require major adjustments to day-to-day clinical practice environments suggest studies of their trainability and efficacy are warranted.

A second grouping of strategies that medical professionals reported using as a means to maintain compassion was possibly broader—maintaining compassion via general self-care. Thus, at least as indexed by these self-report data, healthcare professionals view general physical, social, and psychological care for the self as central to the ability to maintain compassion in clinical practice. Reviews have suggested that self-care may be the most significant preventive measure needed to prevent compassion fatigue (Sorenson et al., 2016), and practical guides to compassion in healthcare treat self-care as the cornerstone of compassion fatigue prevention (Baverstock and Finlay, 2016). Such a finding is likewise consistent with evidence linking self-compassion to health promoting behaviors such as better diet (Dunne et al., 2018), stress management (Allen and Leary, 2010), interpersonal relationships, nutrition, and physical activity (Homan and Sirois, 2017). Interestly, in the empathy literature, self-related thoughts have been suggested to aid in the ability to connect with another individual and take on their perspective (Davis et al., 2004; Gerace et al., 2013). Consistent with views suggesting that compassion fades when those working in healthcare are burnt out or heavily fatigued (Neville and Cole, 2013), self-care featured heavily in the strategies reported by this sample. In some views, compassion evolved to both protect oneself and facilitate cooperative relationships (Keltner, 2009; Strauss et al., 2016). Thus, the maintenance of compassion may also be facilitated by self-care behaviors because they allow the individual to husband resources and, in doing so, provide compassionate care for others. Clearly, such strategies have the potential to be of benefit in the pressured work environments characterizing modern healthcare.

Perhaps unsurprisingly given the prompts used to elicit reports of strategies, the present study revealed that strategies to maintain compassion tended to focus on the self rather than the patient. This finding may suggest an intuitive understanding among healthcare professionals of their own role in the generation of compassionate responses. *Prima facie*, it might suggest that encouraging or training healthcare professionals to engage in self-focused strategies (e.g., mindfulness, tolerance of own discomfort, and self-care behaviors) may improve their ability to not only maintain compassion but also to provide more compassionate care. Although the evidence base is weak and few studies have tested potentially compassion-enhancing interventions in healthcare workforce samples, such interventions might include compassion training (Condon et al., 2013), self-compassion (Rao and Kemper, 2017), Mindfulness-Based Stress Reduction (MBSR; Birnie et al., 2010), meditation (Jazaieri et al., 2013; Weng et al., 2013; Boellinghaus et al., 2014), or clinical supervision (Lindahl and Norberg, 2002).

However, it should also be recalled that the professional is likely only one factor in the process by which compassion is (and is not) generated in healthcare (Fernando et al., 2016). Experimental studies, for example, suggest that the effects of patient characteristics on compassion are often substantially larger than the effects of physician factors (Fernando and Consedine, 2017), perhaps particularly in less experienced practitioners (Reynolds et al., 2019). Patient factors such as the degree to which are seen as responsible for suffering, have off-putting or disgusting characteristics, are unpleasant, rude, demanding or hostile “suck the oxygen” from compassion (Fernando et al., 2016). Thus, although interventions to increase the ability or motivation of healthcare workers to maintain compassion are needed, we must also remember that compassion is a systemic problem that requires systemic solutions.

Overall then, this sample of healthcare professions reported a range of self vs. other, internal vs. external, and immediate vs. general strategies. Although sample selectivity and the specific assessment methods must be borne in mind, the relatively low rate of uncodable responses suggest many compassion-maintaining strategies can be organized along these three underlying dimensions (see Table 4). While some specific strategies (e.g., mindfulness) may periodically reflect both immediate, self-focused, internal strategies as well as internal, self-focused, and *general* strategies, literatures such as those in coping suggest there is utility both in the provision of typologies as well as in considering a professional’s functioning as involving a portfolio of compassion enhancing tools that may be more or less well-developed or employed. A working typology for the strategies reported as maintaining compassion in healthcare is provided in Table 4.

**TABLE 4:** Preliminary typology of self-reported strategies to maintain compassion (with examples).

Temporal range	Focus of strategy			
	Self		Patient	
	More internal	More external	More internal	More external
More immediate	Distress tolerance, imagining patient as family member, awareness of own responses	Go outside and take a breath	Active empathy and perspective taking, remind myself of similarities and our shared humanity, connect with person in front of me, minimize judgment	Active listening, ask patient about their lives, communication skills, consciously warm my eyes or touch patient kindly, use reminders to care
More general	Be a mindful person, practice self-compassion	Self-care including diet, exercise, yoga, meditation, supervision, manage schedules, maintain clinical knowledge, connect with nature, prayer	Remember why I got into this line of work and that it is a privilege to work and learn from patients	

### Characterizing the *Range* of Compassion-Maintaining Strategies

As this project unfolded, it became clear that while some professionals reported a range of strategies encompassing different types of strategy, others reported strategies that were concentrated in particular areas. Because quantifying the tendency to concentrate strategies in different areas may help identify imbalances in a “portfolio” of compassion-maintaining strategies, the *proportions* with which participants’ strategies were concentrated in particular classes of strategy were descriptively assessed. An interesting pattern was evidenced. Specifically, there was a broad three-way split between persons (a) using only self-focused strategies, (b) using some self-focused strategies, and (c) not reporting self-focused strategies. A similar pattern was evident in analysis of the proportions of immediate vs. more general strategies but not for internal vs. externally focused; comparatively few reported only externally focused strategies.

Although it is empirically understudied, the notion that flexibility is central to coping (Cheng et al., 2014), emotion regulation (Aldao et al., 2015), and psychological functioning in general (Kashdan and Rottenberg, 2010), is increasingly accepted. In coping research, for example, it has been shown that having a “balanced” repertoire of coping skills that can be deployed in response to varying environmental demands predicts better psychological adjustment (Cheng et al., 2014). In the context of compassion in health, this approach may suggest that identifying professionals who rely heavily on particular classes of strategy may be of benefit. While any benefits associated with having a “balanced repertoire” needs empirical investigation, it may be that professionals whose compassion maintenance repertoire is unbalanced may struggle to maintain compassion when particular classes of patient or situation are encountered.

The Transactional Model (Fernando and Consedine, 2014a) suggests that compassion arises out of the dynamic interactions between patient, clinician, the clinical picture, and the environments in which compassion is needed. Consistent with this view, basic science work tells us that “compassion fade” is moderated by dispositional factors (Markowitz et al., 2013) and that the extent to which suffering is self-inflicted (Reynolds et al., 2019) and that greater self-other similarity are likely important (Oveis et al., 2010). More directly, experimental studies in health contexts have shown that compassion and patient engagement are more impacted by aversive patient characteristics among student physicians than among more experienced practitioners (Reynolds et al., 2019). Although developmental effects were not evident in the data presented here, individual differences in the tendency or ability to use particular types of compassion-maintaining strategies may well help us understand responses to the array of challenges to compassion evident in healthcare settings. While we do not yet know whether these (or other) strategies are actually effective in maintaining compassion in healthcare settings, providing professionals with an array of options to strengthen or supplement the approaches they typically use is a useful starting point for both research and practice.

### Exploring Possible Developmental Variation in Normative Strategies

Finally, given evidence that compassion fatigue is reliably lower in older/more experienced healthcare professionals (Dev et al., 2019) while compassion satisfaction is greater (Gleichgerrcht and Decety, 2014), a final, exploratory aim of this study was to examine whether the types and proportions of compassion-maintaining strategies reported varied as a function of age. Identifying age-related variation has the potential to identify the strategies that accumulated experience suggests are effective in maintaining compassion over time and thus provide targets for medical education as well as compassion-enhancing interventions.

In this preliminary study, however, age related differences in self-reported strategy use were not evidenced in either differences in the reporting of specific strategies or in the proportion of different types of strategy. Given developmental trends where compassion fatigue and the barriers to compassion appear to lower with age (Dev et al., 2019), our sense here is that developmental variations are likely minimized or obscured in a sample that self-selected for interest in compassion (participants were registered attendees at a compassion conference) and/or that our data were unsuited to providing the necessary “resolution” to find differences. Indeed, the possibility that there is something to learn from the compassion-maintaining strategies that are time-tested in the repertoire of more experienced physicians remains worth pursuing. Age brings with it a wealth of psychological changes (e.g., the tendency to regulate emotions anticipatorily), and covaries with professional experience, seniority, and autonomy. More experienced practitioners report lower barriers to compassion and prior work has shown that a history of past adversity (albeit not necessarily health-related) can enhance compassionate responding under some conditions (Lim and DeSteno, 2016). Given the implications for selection and training in the health workforce, direct examination of this possibility in the context of health is an important future direction for applied compassion research.

### Limitations and Future Research

The present study provides novel insight into the range of strategies health professionals from a range of specialities employ when seeking to maintain compassion as well as the breadth of strategies employed. Although it represents a useful beginning to work in an area with almost no research, there are several limitations that should be borne in mind. First, the sample was self-selected from a group of professionals enrolled to attend the Compassion in Healthcare Conference in New Zealand, March 2019. Although this sample was professionally diverse, those electing to attend this event likely differ from the general population of professionals. Potentially exaggerating self-selection biases is the fact that a direct invitation from study organizers was used. Although participation was anonymous, compassion studies in medicine are prone to bias (Fernando et al., 2017), and both social desirability and self-presentational biases may be important. Thus, the sample may either be (or present) as kinder, more aware of the role of compassion in their work, or be more likely to report particular strategies such as mediation, reference to shared humanity and the like. Equally, the large proportion of self-focused strategies may reflect the phrasing of the specific questions that were asked. Although self-report is likely a necessary first step in this area, such considerations mean the relative frequency with which different types of compassion-maintaining strategies are used among healthcare providers more widely remains unclear.

As importantly, degrees of clinical experience were only indirectly measured via self-reported age. In part, this decision reflects the difficulty in gauging what “clinical experience” actually means and, in part, it reflects the fact that clinical experience and age are so highly correlated to make possible differences empirically near-inseparable (see e.g., Dev et al., 2019). In a similar vein, it is possible that there may be particular aspects of clinical experience that are relevant or that working in different clinical environments lends itself to the use of particular strategies. For example, brief walks to clear the mind and refresh may be more likely/viable when tasks are scheduled in particular ways, where autonomy is greater, and/or where an environment suited to walking is available.

Perhaps most importantly, however, this study does not provide evidence that these self-reported strategies are actually *effective* in maintaining compassion. The fact that strategies are subjectively experienced as helping maintain compassion seems clear but research has yet empirically determine whether subjectively useful strategies translate into a patients experience of greater care. Mindfulness, for example, which was mentioned by a significant minority of participants in this study, has complex links with feelings of compassion as well as with compassionate behavior (Fernando et al., 2016). If the study of compassion in medicine is to further the deployment of compassion-enhancing strategies into education and professional practice it must move beyond ideology, values, and appearance to become an evidence-based agenda. Empirical study evaluating the efficacy of particular strategies is clearly warranted.

## Conclusion

While compassion is increasingly seen as central to patient outcomes as well as work satisfaction among healthcare professionals, prior work has concentrated on the study of compassion fatigue and/or the barriers to compassion. In contributing to this critical applied area of compassion research, the present study presents a first attempt at identifying and descriptively characterizing the strategies health professionals report using as a means to maintain compassion. While some strategies were focused on the patient, these data suggest that self-focused strategies predominate. In turn, such a pattern suggests that self-care is seen as central to the capacity to maintain care for others, something also evident in other writings (Sanchez-Reilly et al., 2013). That said, a significant proportion of the strategies reported were patient-focused, emphasizing empathy, shared humanity, and connecting with patients. Overall, these descriptive data provide initial grounds for moving research beyond a focus on the factors that may interfere with compassion to include the study of the processes that may enhance and sustain it over time. It thus provides fertile grounds for future research to develop and assess interventions designed to maintain medical professionals’ compassion, ensuring the preservation of compassion in healthcare.

## Data Availability Statement

The datasets presented in this article are not readily available because: participants consented to participate with the understanding that access to data would be restricted to the named researchers. Requests to access the datasets should be directed to NC, n.consedine@auckland.ac.nz.

## Ethics Statement

The studies involving human participants were reviewed and approved by the Human Participants Ethics Committee, University of Auckland. The patients/participants provided their written informed consent to participate in this study.

## Author Contributions

AF and NC designed the study and collected the data. SB and VD developed the coding system and coded the data. SB analyzed the data and drafted the initial manuscript. NC oversaw all elements of the project and edited drafts. All authors provided feedback on manuscript drafts and have approved the final version of the manuscript.

## Conflict of Interest

These data were gathered among registered attendees at the Compassion in Healthcare NZ 2019 conference organized by AF and NC. The organizers receive no compensation from this event and any profits are administered in the service of compassion research by the host university. The remaining authors declare that the research was conducted in the absence of any commercial or financial relationships that could be construed as a potential conflict of interest.
