# Supplementary material for: How Do Health Professionals Maintain Compassion Over Time? Insights From a Study of Compassion in Health
Source: Front Psychol. 2020 Dec 29;11:564554. doi: 10.3389/fpsyg.2020.564554 (PMC7802760; doi:10.3389/fpsyg.2020.564554)
Supplement: Supplementary file 1 [file Table_1.docx]

Appendix A

**Strategies To Maintain Medical Compassion Coding Guide**

Version 5.0

November 27^th^, 2019

**Sophie I. Baguley, Nathan S. Consedine, & Vinayak Dev**

**Introduction**.

This coding system is designed to distinguish and organize reports of the strategies medical professionals report using to maintain compassion for their patients. In the initial development study, approximately 150 healthcare professions (including nurses, doctors, psychologists, and other medical professionals) were asked to report the strategies they use to maintain compassion toward their patients. This coding system is designed to identify different classes of strategies health professionals use to maintain compassion.

As a coder, your job is to determine whether each of the coding criteria is met by the information in the text description provided. Most codes are simple “absent-present” codes and are indicated by the numbers “0” and “1”. Others are not, and have a *gradation* of values associated with them. Still others require that you use as many codes as is necessary or as the text satisfies. Most responses will refer to multiple aspects (elements) of the original text and each of these response elements requires its own code.

Although you must understand the system in its entirety to use it most effectively, there is a general comment/rule that apply across categories: You must remember that the purpose of the coding system is make sense of the responses and categorize the different strategies medical professional report. Put another way, you are coding the specific strategies they report, not the irrelevancies or other musings they occasionally record.

The current version of the coding scheme has 7 categories. Some of the categories will appear to have overlap, but you should not concern yourself with this. Each written response must be coded under every code. Oftentimes this will mean putting a “not applicable” code and there will be times when you wonder how a given code is relevant. Again, do not worry about this. Simply make sure that all the narratives from all participants are scored on all codes.

**CATEGORIES**

**I.** **Reference to Connecting with the Patient (CP)**

The “Connecting with the Patient” (CP) codes are intended to identify responses in which the strategies medical professional refers to involve use of connection with the patient. The coding category has two levels and you should code both levels where applicable. Responses are to be coded on a “present-absent” basis in which a “1” is given when the particular code is present in the response and a “0” where it is not. Note: Each response can receive more than one code in this category.

**1. Reference to Communication skills (CP1)**

Definition: This code should be used where reference has been made to the use of communication skills as a means to maintain compassion for patients.

Examples: The text may reference the use of various communication skills such as “open-ended questioning”.

**2. Reference to Active listening (CP2)**

Definition: This code should be used where reference has been made to active listening to maintain compassion.

Examples: The text may reference the use of active listening skills such as “I put down my pen and listen”.

**II. Reference to Common Humanity (CH)**

The “Reference to Common Humanity” (CH) codes are designed to evaluate the elements of a strategy to maintain compassion for patients relating to common humanity (relatedness to one another).The coding category has two levels that build on each other. Every response must be coded on both levels. Responses are to be coded on a “present-absent” basis in which a “1” is given when the particular code is present in the reason for their choice and a “0” where it is not.

**1. Reference to commonality in general (CH1)**

Definition/Criteria: This code should be used when the text involves reference to the shared or common nature of being human.

Examples: The text may reference strategies involving “we are all one”, “we are more similar than different”.

**2. Reference to the commonality of *suffering* (CH2)**

Definition/Criteria: This code should be used when the response includes reference to the commonality of suffering.

Examples: The text may reference to “we all suffer or “all people suffer”.

**III. Reference to Empathy (EP)**

The “Reference to Empathy” (EP) codes are designed to identify reports of empathy as a means to maintain compassion for patients. The coding category has three levels and every response must be coded on all three. Responses are to be coded on a “present-absent” basis in which a “1” is given when the particular code is present in the reason for their choice and a “0” where it is not. Note: Each response can receive multiple scores in this category as a reason can contain the reference to several aspects of empathy.

**1. Reference to openness or connection (EP1)**

Definition/Criteria: This code should be used either when explicit reference has been made to remaining “open” to patients or when the text indicates a more general openness to the experience of others.

Examples: The text may reference “deliberately or consciously open”, “open to them”.

**2. Reference to cognitive empathy or perspective taking (EP2)**

Definition/Criteria: This code should be used when reference has been made to taking the patient’s perspective.

Examples: “I put myself in their shoes”, “I try to imagine their situation from their perspective”.

**3. Reference to affective empathy (EP3)**

Definition/Criteria: This code should be used when reference has been made to *emotionally* connecting with patient distress.

Examples: The text may reference to “feeling moved by the person suffering”, “I connect with my heart”.

# IV. Reference to Processes Emphasizing Tolerance or Management of Psychological Discomfort (TD)

The “Tolerance of Discomfort” (TD) code is intended to identify strategies in which medical professionals seek to maintain compassion via tolerating or manage their discomfort when faced with patient distress. Responses are to be coded on a “present-absent” basis in which a “1” is given when the particular code is present in the response and a “0” where it is not.

Definition: This code should be used when the response references either *internal/external*  psychological processes aimed at increasing their ability to tolerate or manage distress, difficult feelings, or discomfort.

Examples: The text may indicate the processes such as “I notice when I experience fear, distress etc.” “I remind myself it is not their fault they are driving me nuts”. Can also indicate behavioral regulation i.e. “isolation from the situation”, “take a walk when it gets too much”.

**V. Reference to Mindfulness (MS)**

The “Reference to Mindfulness” (MS) code is intended to identify strategies which reference the use of mindfulness to maintain compassion toward patients. Responses are to be coded on a “present-absent” basis in which a “1” is given when the particular code is present in the response and a “0” where it is not.

Definition: This code should be used when general reference has been made to mindfulness as a tool/strategy or a way of being/state of mind to maintain compassion for patients.

Examples: e.g. “I practice mindfulness” or “bringing my whole self to the experience”, or “I remain in the present moment”.

**VI. Reference to Self-care / maintenance (SC):**

The “Reference to self-care / maintenance” (SC) codes are intended to identify responses referring to the use of self-care/maintenance as a strategy to maintain compassion. The coding category has 10 levels and every response must be coded on all 10 criteria. Responses are to be coded on a “present-absent” basis in which a “1” is given when the particular code is present in the response and a “0” where it is not. Note: Each response can receive multiple scores in this category as a reason can contain the reference to several self-care/maintenance.

**1. Reference to recognizing limits (SC1)**

Definition: This code should be used when reference has been made to the medical professional recognizing their limits within their realm.

Examples: The text may reference to “I acknowledge that I am human and cannot fix everything”.

**2. Reference to supervision (SC2)**

Definition: This code should be used when reference has been made to using supervision to maintain compassion.

Examples: The text may reference the use of “supervision”, see a “clinical psychologist”, “debrief cases with other clinicians”.

**3. Reference to exercise (SC3)**

Definition: This code should be used when reference is made to using exercise or physical activity as a means of self-care.

Examples: The text may reference to “going to the gym”, or “going for a run”.

**4. Reference to taking time out of schedule (SC4)**

Definition: This code should be used when reference has been made to taking time out of their work schedule to refresh themselves and look after their wellbeing.

Examples: The text may reference to “taking a holiday”, “ensure I reward myself with time off”.

**5. Reference to socialization (SC5)**

Definition: This code should be used when reference has been made to engaging in socialization for self-care/maintenance.

Examples: The text may reference “spending time with friends”, “catching up with people close to me”

**6. Reference to control of schedule/workload (SC6)**

Definition: This code should be used when reference has been made to limiting work schedules to ensure they do not burnout or become exhausted.

Examples: The text may reference to only working “set hours per week”, “take two days off each week”.

**7. Reference to praying (SC7)**

Definition: This code should be used when reference has been made to praying as a means to maintain compassion for patients.

Examples: The text may reference “I speak to God”, “praying for compassion”.

**8. Reference to going to church (SC8)**

Definition: This code should be used when reference has been made to the act of going to church or a religious establishment.

Examples: The text may reference to “attending church” or “visiting my holy place”.

**9. Reference to meditation (SC9)**

Definition: This code should be used when reference has been made to using meditative practices

Examples: Responses include reference to meditation e.g. “I practice meditation”.

**10. Reference to maintenance of other physical and mental self-care (SC10)**

Definition: This code should be used when reference to self-care behaviors that are not able to be captured by the categories above are evident.

**VII.** **UnCodable Response Code (UR)**

The “UnCodable Response” (UR) code is to be used where the participant has failed to provide a meaningful response, where they have written gibberish, or where they have otherwise failed to provide information that can be meaningfully coded in any category including VII (reference to other strategies; OS). Responses are to be coded on a “present-absent” basis in which a “1” is given when the response CANNOT be coded and “0” where it CAN.
